# Calling for a united action to defeat COVID-19

**DOI:** 10.1093/pcmedi/pbaa027

**Published:** 2020-08-06

**Authors:** Madison Overby, Qinqin Pu, Xiawei Wei, Min Wu

**Affiliations:** Department of Biomedical Sciences, School of Medicine and Health Sciences, University of North Dakota, Grand Forks, ND 58202, USA; Department of Biomedical Sciences, School of Medicine and Health Sciences, University of North Dakota, Grand Forks, ND 58202, USA; Laboratory of Aging Research and Cancer Drug Target, State Key Laboratory of Biotherapy and Cancer Center, National Clinical Research Center for Geriatrics, West China Hospital, Sichuan University, Chengdu 610041, China; Department of Biomedical Sciences, School of Medicine and Health Sciences, University of North Dakota, Grand Forks, ND 58202, USA

## Abstract

The widespread and lingering pandemic of COVID-19 is partly due to disjointed international countermeasures and policies enforced by different countries. We have been witnessing disparity in policies and measures in different countries and regions: some are in much better control than others. To effectively deal with this and future devastating pandemics, we as human beings must work together to coordinate a concerted, cooperative international policy to reduce or possibly avoid unnecessary health crises, and life and economic losses.

## Introduction

The greatest difficulties in controlling the spread of COVID-19 are the uncertainty of the incubation period, the large number of asymptomatic cases, and the super transmissibility of the SARS-CoV-2 pathogen.^1^ Because of the lack of specific vaccines and effective drugs at this time, the world will have to fall back on policy changes, supporting medical personnel, continued research into COVID-19, and, probably the most important, fighting the disease in unison; otherwise we may continue to see the multiple waves of pandemic spread from region to region and continent to continent. Ironically, the international attention to the pandemic and levels of countermeasures are extremely varied, which may be attributed to the cause of continued spread. As humans are far more interconnected than decades ago, it only needs a small portion of international communities with irrational approaches to make the job of curbing this disease and future pandemics much more difficult. Here, we attempt to discuss the lack of collaboration, and how we can work in parallel internationally to avoid further devastating damage and contain the pandemic as soon as possible.

## Divergent policies may accelerate and intensify COVID-19 spread

Policy changes implemented by central governments have been the single most effective way of controlling the spread thus far. “Flattening the curve” has become a household term, which emphasizes the effect of staying home and preventing mass spread of SARS-CoV-2 to decrease both infection rates and mortality rates. China was one example, especially of Asian countries, that was able to lower transmission rates to almost zero in the early months. They did this by enforcing the strictest isolation strategy: the suppression strategy.^2^ “Traffic control, limitation of travel, extension of the Chinese New Year vacations, delay of returning to work, rigorous management of communities and even the wartime management have ensured that the susceptible population stay home”.^3,4^ More countries are going to need to look toward the strict governmental action of China to get the virus under control sooner rather than later and prevent the potential “second wave and even multiple waves”. The suppression strategy adopted by China and other Asian countries such as South Korea, Japan, and Singapore is in contrast to the mitigation strategy that is most commonly executed in the western nations. The goal of the mitigation strategy is to slow the spread of the disease and mitigate the effects of the virus on healthcare systems, as well as the social perception of the epidemic, whereas the suppression strategy aims to reduce the transmission rate (*R*_0_) to less than one, which essentially reverses the epidemic.^2^ Policy implementations thus far are the only way all people will stay home and commit to social distancing. This was also seen more recently in Spain, which was originally projected to have a similar outcome to the high mortalities of Italy.^5^

Spain hit its peak in late March to early April (ECDC, 2020). It capped out on new cases on 27 March with a reported high 9181 new cases reported by the ECDC (ECDC, 2020). The death toll hit its maximum just a few days later on 3 April at 950 new deaths related to COVID-19. The government in Spain began a mitigation strategy on 14 March, after new cases were already in the exponential growth phase.^2^ With the late start to containment of the virus by governmental action, Spain was soon ranked second in case count behind the United States and second in deaths behind Italy by 6 April.^2^ However, as cases continued to rise, lockdown orders became stricter as the President “demanded unity and social responsibility”.^5^ After lockdown orders were issued on 18 March, cases began to dwindle.

Italy also missed the opportunity for an early response, which may be the potential cause of a relatively severe pandemic in the European regions. In early March, Italy became the first western country to launch a nationwide lockdown to contain the SARS-CoV-2 outbreak, but, despite these strict measures, the number of cases continued to rise. Italy's universal healthcare system is among the best in industrialized countries, and it is a top producer of many types of medical equipment and supplies. However, it was already too late, as hospitals and medical staff were overwhelmed, prompting anguished debate regarding how to control this emerging and violent virus.

Relatively early on (28 February 2020), most cases in Italy were in the Lombardia and Veneto regions of Northern Italy. However, cases were later escalating at an alarming rate with 30 000 cases reported and 3405 deaths as of 19 March 2020. Geographic lockdown and social distancing failed to significantly block the spread of this horrible virus.

If Italy had implemented these measures in early February, it may have been a different scenario. This is true for the US, the UK, Spain, and other countries. Many of these industrialized countries were not alert enough early on and waited too long before implementing a final shutdown or lockdown. The inability to respond swiftly to a very tough, peculiar virus such as this one has really educated us as human beings on how we should prepare right now and react during future pandemics. When the Chinese authorities locked down the land to stop the spread of the disease, we as international communities should have paid extra attention to avoid the possibility of insurmountable spread resulting in a downturn in almost every facet of life including travel, dining, gathering, working, studying, and even research into the causal pathogen SARS-CoV-2 virus.

The biggest question as to why Italy was hit the hardest in the March–April period, while so many other nations that reported their first cases at the same time in February have not experienced such a terrible situation, remains to be fully answered. Dr. Remuzzi, a physician at an Italian hospital, heard the following from general practitioners: “The very strange and severe pneumonia has been around in the northern region such as Lombardy in December and even November, particularly frequently seen in old populations, like COVID-19”.^6^ This information has not been confirmed with other reports. However, had the Italian authorities taken strong action earlier after seeing the first case at the end of January 2020, the situation would have not been so devastating. This suggests that the Italian nation may have been suffering the problem without sufficient awareness before the disease progressed to being almost uncontrollable.

## Policy changes are required to combat this type of pandemic

Policy changes and an increase in social distancing will also ensure that medical personnel and supplies are not exhausted. The key to a successful pandemic response is control of medical supplies. There needs to be an abundance of supplies in reserve in the case of disease outbreak as well as sufficient allocation of emergency medical supplies.^7^ An example of what can happen without proper preparedness can be seen in Pakistan. Pakistan had “no contingency plan, no training to deal with healthcare emergency, hospitals were not designed to deal with a pandemic down to their fundamentals”. This has led to trouble containing the virus in the country, where cases continue to increase (ECDC, 2020). According to Salman *et al*. (2020), only 60% of respondents to an economic survey of Pakistan have access to personal protective equipment (PPE).^8^

The challenge for policy change is to make an urgent, unified, and internationally coordinated effort to counteract an emerging pandemic, which is lacking at the moment. Due to the currently disorganized actions, people have witnessed COVID-19 waves in multiple areas, countries, and continents, which seems impossible to control in a timely manner and may result in continued and lingering recurrences. This is compounded by the mutations of the SARS-CoV-2 virus, which seems to be gaining strength during the spread. As a typical example, the small spike in Beijing from 11–16 June 2020 was possibly a reentry from Europe, appearing more lethal than the original Wuhan strain.^9^ In addition, some continents such as Africa and South America have recently seen a surge in case reports and deaths, while their governments were idle during the first wave in Asian countries. Moreover, the second wave in southern states of the USA, such as Florida and Oklahoma, were due to the less coordinated policy from the US Government and state governing bodies (Johns Hopkins University, 2020). The non-unified countermeasures are the biggest culprits of the continued severe pandemic. There are many other matters that need to be tackled on a global scale such as medical supplies and vaccine research. These problems are calling for unified activities and more concerted measures to be taken in future. However, there is a lack of leadership and governing bodies in the world. We strongly suggest that the United Nations, or probably a new organization, will need to draft and coordinate internationally agreeable policies, activities, and measures to effectively combat this as well as future pandemics.

Sufficient stocks and fast production of medical supplies such as PPE protect not only patients in the already overcrowded hospitals, but also other populations in large. This also directly impacts the number of medical personnel that are infected with SARS-CoV-2 while working with these patients. In the Lombardy region of Italy, which was faced with extreme shortages of both medical supplies and workers, 20% of healthcare workers contracted COVID-19 during their attempt to diagnose and treat patients.^6^ The situation in Lombardy got much worse before it got better, where medical personnel were being forced to distribute supplies based on personal discretion, deciding which patients were more likely to live or die with the assistance of medical support.^6^ In contrast to the situation in Lombardy, a hospital in Belgium had a high availability of PPE, high standards of infection prevention, PCR screening for symptomatic staff, and focused intensely on contact tracing and quarantine.^9^ One hospital in Belgium tested 3056 hospital employees, and 197 people tested positive for IgG antibodies for SARS-CoV-2, which is approximately 6.4%.^10^

Finally, testing needs to be rapid and accurate to slow the spread of the virus both now and in the future. The high amount of asymptomatic cases makes this absolutely necessary. In order to return to normal life, people will have to be tested regardless of whether they are experiencing symptoms or not, which at this moment in time is not possible.^3^ Early diagnosis, isolation, and treatment would ensue. This strategy of testing the majority of a population has seen success in South Korea. As of 26 February 2020, the average reproductive rate in Korea was determined to be 1.5.^10,11^ As a result of early widespread testing and an extremely fast social distancing response put in place by the government, the basic reproductive rate was kept to less than half of the international average. This very clearly shows the importance of a fast and thorough response in the eye of a pandemic storm.

## Global collaborations facilitate development of pharmaceutics and vaccines

We know that during the current time international collaboration is a great trend and may be the only path to develop effective drugs and vaccines to conquer the pandemic. It is a positive response from some of pharmaceutic and philanthropic entities to bring together the dollars and workforces to more quickly discover and validate effective therapeutics or prevention measures. The Coalition for Epidemic Preparedness Innovations (CEPI) is a foundation with support from all sources and has shown promise in its short three-year history. Humanity will continue to foster this type of organization with more government involvement to expand efforts and funding levels. We believe that there will be bright future in front of us for vaccines and drug research.

There are still many challenges before us. The European Commission and the World Health Organization (WHO) organized the Global Vaccination Summit last September (2019) to prepare a roadmap for future pandemics, focusing on the following tasks in vaccine research (WHO website, https://www.who.int/news-room/events/detail/2019/09/12/default-calendar/global-vaccination-summit). The aim is to improve international political leadership, commitment to vaccination, and building effective collaboration and partnerships across international, national, and all levels with health authorities, health professionals, civil society, communities, scientists, and industry. These steps will enable the prevention and treatment of emerging diseases everywhere through sustained research and development to provide timely delivery to every person for all epidemics and pandemics. Humans have to promote vaccine immunization for all countries. In addition to this, vaccine regimens should be implemented and financial sustainability strengthened on broad scales to cover everyone. Concerted efforts would help build strong surveillance systems for vaccine-preventable diseases, such as coronaviruses. Considering 18 years have passed since the SARS-CoV-1 outbreak, we should feel the urgency to produce effective therapeutics and preventive vaccines at an accelerated pace. It is encouraging to see multiple vaccines currently rapidly entering phase III clinical trials for COVID-19.^11–14^ New technology will be reinforced to monitor the performance of vaccination programs. Most importantly, we must sustain research efforts to invent, develop, and validate new vaccines and drugs based on the evidence. Outsources will be actively explored to seek investment, including novel models of funding and incentives, in research, development, and innovation for new or improved vaccine and delivery devices.^3^ This can help reduce vaccine shortages through improved vaccine availability monitoring, forecasting, purchasing, delivery, and stockpiling systems, and collaboration with producers and all participants in the distribution chain to make the best use. Importantly, we need to help rid people of vaccine hesitancy, increasing confidence in vaccinations, as well as designing and implementing evidence-based interventions. We need to encourage healthcare professionals to provide effective and objective information to the public and fight misinformation in normal and social media platforms. In short, international communities will have to create and integrate vaccination in global health and develop agendas through continued efforts.

Drugs are probably the most effective weapons to combat emerging diseases. Currently, there is intense interest in development and production of pharmaceuticals for COVID-19. However, the main defect in this industry is the reluctant investment and efforts to promote an attitude of preparedness during normal times. During the SARS-CoV-1 spread, the government as well as the vaccine and drug industry invested a significant amount in research and development; however, the levels of enthusiasm faded quickly. This experience teaches us that this should not happen again. Currently, we have no idea when the next emerging or emerged diseases will revisit humans. Preparedness is a great way to control diseases we do not see but can only imagine, such as the current pandemic. Coronaviruses are the one of examples, but many other pathogens such as Zika virus, Ebola virus, and some types of influenza virus should be carefully targeted. In fact, remdesivir, an investigative drug originally developed for Zika virus by Gilead Sciences Inc., has shown promise in treating COVID-19.^12,14,15^ Hence, we have done some decent work but increased efforts are urgently needed to fight against future diseases. The better news is that many pharmaceutical companies will rethink the strategies to prevent and treat the next pandemics. An interactive and collaborative example in the European Union, the EMERGE (efficient response to highly dangerous and emerging pathogens at EU level) program, provides another excellent opportunity to work in unison to combat emerging and re-emerging infectious pathogens with the potential to cause serious cross-border outbreaks. The Joint Action EMERGE comprises a European network with about 40 diagnostic laboratories focused on risk group 3 bacteria and risk groups 3 and 4 viruses, which has facilitated research toward developing a better disease control infrastructure and a working relationship with so many top scientists in related fields. We need to have more of EMERGE in every continent and ideally every country.

## Concluding remarks

In summary, we have discussed the strategies and countermeasures from global levels to combat the insurmountable, devastating, lingering COVID-19 pandemic. The single most important factor is policy changes, which may evolve with time, but should be based on solid and up to date scientific evidence, other nations’ successful examples, and experts’ opinions from international communities rather than individual leadership preference, cultural variation, and political opinion. Second, social distancing is widely applied internationally as a moderate means to curb the disease spread but requires high levels of individual cooperation. As the complete lockdown is highly effective to rapidly stop spreading, it may be taken into consideration if necessary in the future. Third, personal protective equipment (masks, gloves, etc.) and medical equipment such as ventilators are critical for flattening the curve and should be produced and stocked in sufficient levels as soon as possible when a potential pandemic begins, even if not reaching home soil. Fourth, testing early and on a large scale appears very important in isolating and blocking unnecessary dissemination of the disease. In conclusion, to effectively battle this and future cruel pandemics, we as human beings should all work together to coordinate a highly concerted international policy by a higher authority (that may need to be created) to deal with the many uncertainties and reduce or possibly avoid unnecessary health crises, and life and economic losses.

## Authors’ contributions

M.O. and M.W. wrote the manuscript. Q.P. and X.W. made comments. Q.P., M.O., X.W. and M.W. revised the manuscript.
